# Development and validation of machine learning models for 30-day mortality prediction in septic shock

**DOI:** 10.3389/fmicb.2026.1908032

**Published:** 2026-08-28

**Authors:** Shanbi Chang, Yuebang Wang, Zijin Zhang, Jian Sun, Juan Wu, Dan Wang, Wenyang Hou, Jun Zhou, Baoyu Wang

**Affiliations:** 1 Department of Clinical Medicine, Jiangsu Province (Suqian) Hospital, Suqian, Jiangsu, China; 2 Core Lab (CL), Roche Diagnostics (Shanghai) Limited, Shanghai, China; 3 Department of Laboratory Medicine, The First Affiliated Hospital with Nanjing Medical University, Nanjing, Jiangsu, China; 4 Department of Clinical Medicine, Xuyi People's Hospital, Huaian, Jiangsu, China

**Keywords:** 30-day mortality, machine learning, model, prediction, septic shock

## Abstract

**Background:**

Septic shock carries a high risk of death, yet traditional prognostic tools have limitations. This study aimed to develop and validate machine learning (ML) models for predicting 30-day mortality in septic shock patients using comprehensive admission variables.

**Methods:**

A retrospective analysis was performed on 695 patients with septic shock. Demographic characteristics, vital signs, and 33 laboratory parameters were included as candidate predictors. LASSO regression was used for feature selection from age, gender and 33 laboratory parameters. Eleven ML algorithms were developed using a two-way split with internal cross-validation (60% training with 5-fold CV for hyperparameter tuning, 40% held-out validation cohort for final evaluation) and their performances were evaluated using AUC with 95% confidence intervals (CIs), sensitivity, specificity, calibration curves, and decision curve analysis. SHAP method was applied for model interpretation.

**Results:**

LASSO selected six predictors: lactate, platelet count, neutrophil percentage, monocyte percentage, albumin, and C-reactive protein. Following rigorous hyperparameter tuning, the Random Forest (RF) model demonstrated the best and most stable performance in the validation cohort (AUC = 0.820, 95% CI: 0.765–0.875). The RF model demonstrated good calibration and net clinical benefit. A web-based risk calculator was subsequently developed.

**Conclusion:**

The RF model based on six readily available laboratory parameters accurately predicts 30-day mortality in septic shock patients. However, the findings are limited by the single-center retrospective design and lack of external validation, warranting further multi-center studies to confirm generalizability.

## Introduction

The most severe phase of sepsis progression is septic shock, whose pathophysiological hallmark is infection-induced circulatory failure and metabolic abnormalities (Meyer and Prescott, 2024). Even with adequate fluid resuscitation, patients often require vasopressor support to maintain blood pressure and frequently exhibit persistent hyperlactatemia. As defined by the Third International Consensus Definitions for Sepsis and Septic Shock (Sepsis-3), septic shock carries a substantially higher mortality risk than sepsis alone (Shime et al., 2025; Seymour et al., 2016; Jouffroy et al., 2024). Therefore, early and accurate prediction of 30-day survival has become a critical priority in intensive care, aimed at optimizing treatment strategies and allocating medical resources rationally.

Traditional prognostic tools, such as the Sequential Organ Failure Assessment (SOFA) score and the Simplified Acute Physiology Score II (SAPS-II), are widely used in clinical settings but have limitations in predictive performance (Dao et al., 2025; Lambden et al., 2019). These scoring systems often fail to adequately integrate and leverage the large volume of multidimensional laboratory data collected shortly after patient admission—including blood lactate, platelet count, and white blood cell differentials. Such parameters carry essential information that reflects disease severity and prognosis (Klemm et al., 2024; Hernández et al., 2019; Choi et al., 2025; Clere-Jehl et al., 2019). Consequently, relying solely on conventional methods cannot meet the heightened demands for prognostic accuracy in the current era of individualized precision medicine.

Machine learning (ML), inspired by how the human brain approaches pattern recognition, offers an artificial intelligence-based solution. It addresses classification problems by adjusting the weights of hidden layers during training to minimize an error function (Ong et al., 2012). Machine learning has demonstrated potential in predicting clinical outcomes across various disease states (Hu et al., 2024; Pruthi et al., 2024; Shi et al., 2024). Leveraging these algorithms facilitates the development of valuable prognostic models for patients with septic shock, helping clinicians formulate treatment and management strategies. This study aims to construct and validate 11 ML models using age, sex, and 33 laboratory parameters to predict 30-day mortality in patients with septic shock.

## Methods

### Study design and population

This study was a single-center retrospective cohort study. Consecutive adult patients with septic shock who were hospitalized at Jiangsu Province (Suqian) Hospital between August 2021 and August 2025 were enrolled. The inclusion criteria were as follows: (1) age ≥18 years; (2) meeting the diagnostic criteria for septic shock according to the Sepsis-3 definitions (Seymour et al., 2016) during the hospital (requiring vasopressor therapy to maintain a mean arterial pressure ≥65 mmHg despite adequate fluid resuscitation, and having a serum lactate level >2 mmol/L); (3) completion of laboratory tests, including complete blood count, biochemistry, and C-reactive protein, within 24 h of admission. The exclusion criteria were: (1) Patients with multiple hospitalizations, only data from the first admission were included (13 patients); (2) Patients with loss to follow-up within 30 days or unknown outcome information (8 patients); (3) Patients with a history of chronic end-stage renal disease (requiring long-term dialysis) (11 patients), hepatic failure (Child-Pugh class C) (17 patients), hematologic malignancy, or current chemotherapy/radiotherapy (6 patients); (4) pregnant or lactating women (4 patients). A total of 695 patients were ultimately included in the analysis.

No *a priori* sample size calculation was performed, as this was a retrospective study with consecutive enrollment of all eligible patients. However, the final sample (*N* = 695, with 148 outcome events) provided an EPV of 24.7 (148 events/6 predictors), well above the recommended minimum of 10 for reliable prediction model development.

This study was approved by Jiangsu Province (Suqian) Hospital’s Ethics Committee, and conducted in accordance with the Declaration of Helsinki (Approval No.: 2026-SR-0133). Informed consent was waived due to the retrospective nature of the study.

### Data collection and feature selection

Admission variables were recorded for each patient, including demographic characteristics, vital signs, laboratory parameters, and clinical outcomes. A total of 35 candidate variables (age, sex, and 33 laboratory parameters) were entered into the LASSO regression for feature selection. Demographic characteristics included age (years) and sex. Vital signs included body temperature (T, °C), respiratory rate (R), pulse rate (P), systolic blood pressure (SBP, mmHg), and diastolic blood pressure (DBP, mmHg)- these vital signs were used for descriptive baseline comparison only and were not entered into LASSO. Laboratory parameters comprised complete blood count, including red blood cell count (RBC), white blood cell count (WBC), lymphocyte count (LY), lymphocyte percentage (LY%), monocyte count (MO), monocyte percentage (MO%), neutrophil count (NE), neutrophil percentage (NE%), hemoglobin (Hb), hematocrit (HCT), mean corpuscular volume (MCV), red blood cell distribution width (RDW), platelet count (PLT), mean platelet volume (MPV), and platelet distribution width (PDW); coagulation function, including prothrombin time (PT), thrombin time (TT), activated partial thromboplastin time (APTT), international normalized ratio (INR), fibrinogen (FIB), and D-dimer (D-D); liver function, including gamma-glutamyl transferase (*γ*-GT), alanine aminotransferase (ALT), aspartate aminotransferase (AST), alkaline phosphatase (ALP), lactate dehydrogenase (LDH), total bilirubin (TBIL), direct bilirubin (DBIL), indirect bilirubin (UBIL), total protein (TP), albumin (ALB), globulin (GLB), and albumin-to-globulin ratio (A/G); lipid profile, including total cholesterol (TC), triglycerides (TG), high-density lipoprotein cholesterol (HDL-C), and low-density lipoprotein cholesterol (LDL-C); renal function and electrolytes, including urea (UREA), creatinine (Cr), uric acid (UA), glucose (GLU), calcium (Ga), chloride (Cl), potassium (K), and sodium (Na); and inflammatory and cardiac biomarkers, including C-reactive protein (CRP), procalcitonin (PCT), cardiac troponin I (CTnI), N-terminal pro-brain natriuretic peptide (NT-proBNP), tumor necrosis factor-alpha (TNF-α), interleukin-6 (IL-6), and lactic acid (LAC). All continuous variables were entered into the models using their original raw values without transformation or normalization. The outcome measure was 30-day mortality.

### Model building strategy

All samples were randomly split into a training and a validation set at a 6:4 ratio using scikit-learn (version 1.2.2). Critically, GridSearchCV with 5-fold cross-validation was applied exclusively to the training cohort to optimize hyperparameters, ensuring no information from the held-out validation cohort was used during model development. The validation cohort remained completely untouched until the final model evaluation, providing an unbiased estimate of model generalizability.

First, the Least Absolute Shrinkage and Selection Operator (LASSO) method was employed in the training cohort. LASSO imposes a constraint on the sum of the absolute regression coefficient values, forcing some coefficients to zero and yielding a more parsimonious model. This approach effectively handles datasets with complex covariance structures while retaining the most relevant features. Then, multicollinearity among the LASSO-selected predictors was assessed using the variance inflation factor (VIF). Variables with VIF > 5 were removed. VIF analysis was used as a confirmatory diagnostic tool rather than a primary feature selection method, as LASSO inherently handles collinearity. The remaining variables were then entered into ML. Eleven ML algorithms were developed and validated: Naïve Bayes (NB), K-Nearest Neighbors (KNN), Support Vector Machine (SVM), Logistic Regression (LR), Random Forest (RF), Decision Tree (DT), Artificial Neural Network (ANN), Gradient Boosting Decision Tree (GBDT), Light Gradient Boosting Machine (LightGBM), eXtreme Gradient Boosting (XGBoost), and Adaptive Boosting (AdaBoost). GridSearchCV (scikit-learn 1.2.2) with 5-fold cross-validation was used to optimize hyperparameters for each model. These models aimed to predict 30-day mortality in adult septic shock patients. Based on the selected predictors, the performance of the 11 algorithms was compared to identify the best model.

**Figure 1:**
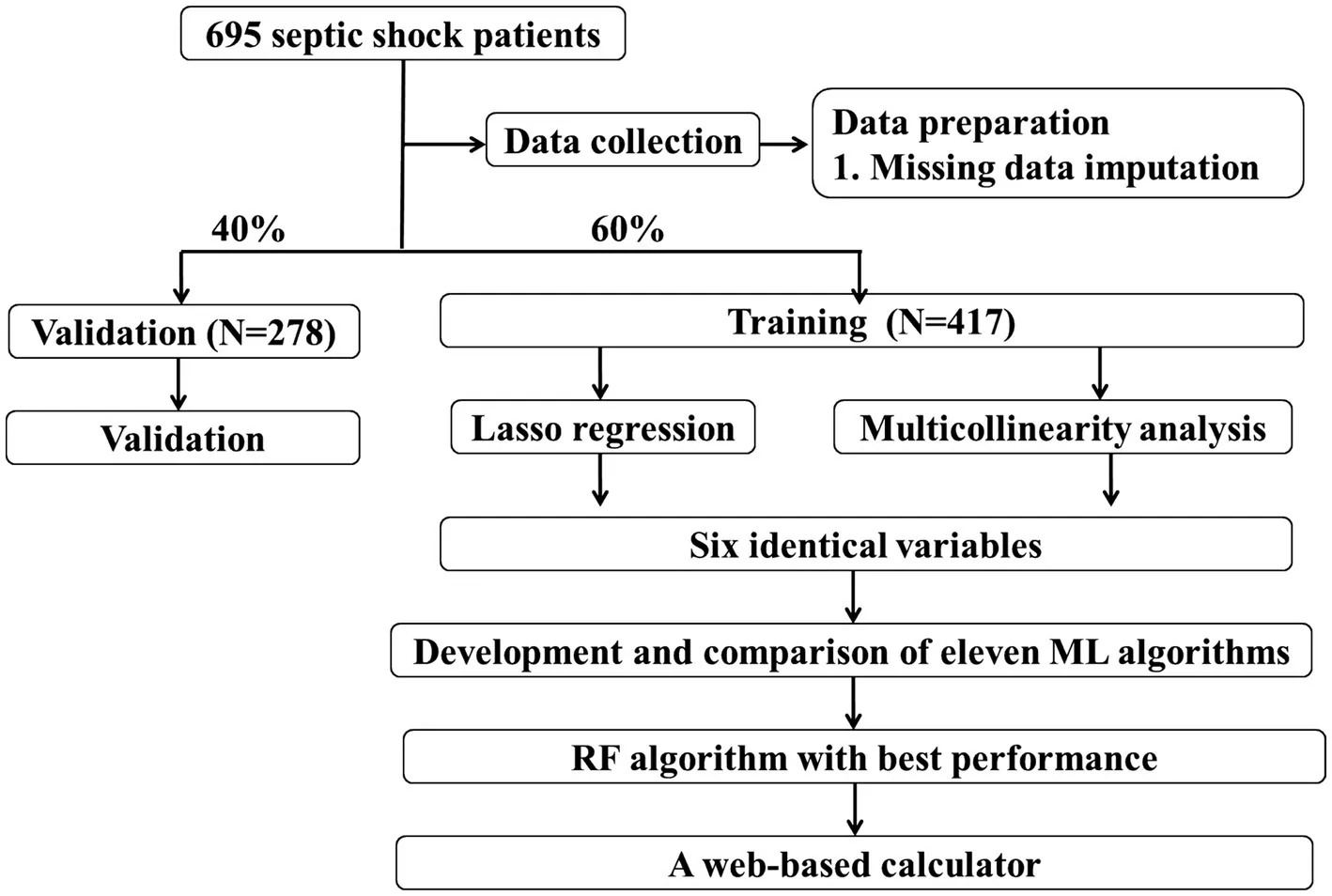
Flow chart of the survey design.

Predictive performance was evaluated using the area under the curve (AUC) sensitivity, specificity, accuracy, positive predictive value (PPV), negative predictive value (NPV), F1-score, calibration curves and decision curve analysis (DCA). All performance metrics are reported with their 95% confidence intervals (CIs). The algorithm with the highest AUC in the validation cohort was designated as the optimal algorithm for subsequent research. Additionally, SHapley Additive exPlanations (SHAP 0.44.0) values were applied to determine each predictor’s importance and to visualize its relationship with 30-day mortality (Figure 1).

### Outcomes

The primary outcome of this study was defined as all-cause mortality within 30 days following the diagnosis of septic shock, Patient prognostic outcomes were obtained from medical records, outpatient follow-up visits, or telephone interviews.

### Statistical analysis

Continuous variables were reported as mean (± standard deviation) or median (interquartile range), and group comparisons were performed using the two-tailed Student’s *t*-test or the Mann–Whitney *U* test, as appropriate. Categorical variables were expressed as counts (percentages) and analyzed with the chi-square test or Fisher’s exact test, depending on the conditions. A two-sided significance level of 0.05 was set for all statistical tests. The machine learning models were developed using the MyLab+i-Research platform. Random forest (RF) imputation (n_estimators = 100, max_depth = 10, and random_state = 42) was applied for variables with less than 5% missing data. Variables [adenosine deaminase (ADA, 90.1% missing), blood ammonia (91.8% missing)] and Serum amyloid A (SAA, 79.4% missing) with 5% or more missing values were excluded from the final analysis to maintain the reliability and validity of the results.

## Results

### Population characteristics

A total of 695 patients were included in this study, of whom 247 (35.50%) died within 30 days. Patients were randomly divided into training and validation cohorts, and baseline demographic characteristics were comparable between the two cohorts (Supplementary Table 1).

In the training cohort, 148 patients (35.5%) died within 30 days. No significant differences were observed between the survival and non-survival groups in terms of age, sex, RBC, Hb, HCT, MCV, ALT, A/G, TC, FIB, ALP, TG, LDL-C, electrolytes (K, Na, Cl), blood pressure, or body temperature (all *p* > 0.05). In contrast, the non-survival group exhibited significantly higher levels of WBC, NE, PLT, CRP, PCT, coagulation parameters (PT, APTT, INR, D-D), liver function markers (AST, LDH, TBIL, *γ*-GT), renal function markers (UREA, Cr, UA), GLU, cardiac markers (cTnI, NT-proBNP), inflammatory cytokines (TNF-*α*, IL-6), LAC, R and P. Meanwhile, LY, MO, MPV, ALB, GLB, HDL-C, and Ca levels were significantly lower in the non-survival group (all *p* < 0.05) (Table 1).

**Table 1:** Comparison of baseline characteristics between septic shock patients with different prognoses in the training cohort.

Characteristic	Overall *N* = 417	Survivor *N* = 269	Non-survivor *N* = 148	*p*-value
Sex (male)	263 (63.1%)	172 (63.9%)	91 (61.5%)	0.619
Age (yr)	70 (59, 79)	70 (59, 79)	71 (59, 79)	0.639
RBC (×10^12^/L)	3.31 (2.80, 3.84)	3.34 (2.90, 3.84)	3.25 (2.69, 3.90)	0.213
WBC (×10^9^/L)	8 (6, 13)	8 (5, 10)	11 (6, 16)	<0.001
LY (×10^9^/L)	0.92 (0.53, 1.35)	1.06 (0.74, 1.49)	0.61 (0.32, 1.03)	<0.001
LY%	11 (6, 19)	15 (9, 22)	6 (4, 11)	<0.001
MO (×10^9^/L)	0.43 (0.26, 0.67)	0.47 (0.34, 0.70)	0.27 (0.13, 0.64)	<0.001
MO%	5.5 (2.8, 8.0)	6.7 (4.7, 8.8)	2.8 (1.8, 4.6)	<0.001
NE (×10^9^/L)	6.4 (3.9, 10.9)	5.6 (3.7, 8.3)	9.7 (5.1, 14.3)	<0.001
NE%	80 (69, 90)	75 (66, 84)	90 (82, 94)	<0.001
Hb(g/L)	98 (85, 115)	99 (85, 114)	95 (82, 116)	0.302
HCT (%)	30.2 (26.4, 35.3)	30.7 (26.7, 35.0)	29.7 (25.7, 36.2)	0.399
MCV (fl)	93 (89, 96)	92 (90, 96)	93 (89, 97)	0.164
RDW (%)	14.60 (13.40, 16.10)	14.40 (13.30, 16.00)	15.05 (13.80, 16.20)	0.039
PLT (×10^9^/L)	172 (89, 255)	219 (153, 293)	78 (39, 154)	<0.001
MPV	10.81 ± 1.40	10.68 ± 1.33	11.05 ± 1.50	0.013
PDW	16.10 (13.20, 16.80)	15.70 (11.90, 16.40)	16.60 (15.10, 17.50)	<0.001
CRP (mg/L)	79 (40, 134)	60 (28, 104)	126 (77, 186)	<0.001
PCT (ng/mL)	3 (1, 16)	2 (0, 10)	12 (2, 29)	<0.001
PT(s)	12.5 (11.5, 14.2)	12.0 (11.3, 13.4)	13.6 (11.9, 17.0)	<0.001
TT(s)	17 (16, 18)	17 (16, 18)	17 (16, 25)	<0.001
APTT(s)	30 (27, 35)	28 (26, 32)	33 (29, 45)	<0.001
INR (Ratio)	1.09 (1.00, 1.25)	1.05 (0.98, 1.18)	1.19 (1.04, 1.50)	<0.001
FIB (g/L)	4.67 (3.40, 6.09)	4.70 (3.57, 6.09)	4.55 (2.44, 6.11)	0.108
D-D (mg/L)	4 (2, 8)	3 (2, 6)	6 (3, 12)	<0.001
γ-GT (U/L)	51 (27, 102)	53 (30, 109)	42 (22, 83)	0.012
ALT (U/L)	25 (14, 44)	25 (14, 43)	26 (13, 51)	0.617
AST (U/L)	30 (20, 57)	28 (19, 46)	38 (22, 110)	<0.001
ALP (U/L)	97 (73, 141)	96 (76, 137)	99 (69, 148)	0.735
LDH (U/L)	240 (185, 358)	209 (175, 279)	330 (234, 612)	<0.001
TBIL (μmol/L)	14 (10, 22)	13 (9, 19)	17 (12, 43)	<0.001
DBIL (μmol/L)	5 (3, 10)	4 (2, 8)	9 (4, 27)	<0.001
UBIL (μmol/L)	7 (4, 11)	7 (3, 10)	8 (5, 15)	0.003
TP (g/L)	58 ± 9	60 ± 8	55 ± 10	<0.001
ALB (g/L)	30.4 ± 5.0	31.6 ± 4.4	28.2 ± 5.3	<0.001
GLB (g/L)	27 (24, 31)	28 (25, 32)	26 (22, 29)	<0.001
A/G	1.13 (0.95, 1.29)	1.14 (0.96, 1.30)	1.09 (0.90, 1.27)	0.125
TC (mmol/L)	2.90 (2.83, 3.14)	2.89 (2.83, 3.32)	2.92 (2.84, 3.08)	0.972
TG (mmol/L)	1.41 (1.16, 1.74)	1.41 (1.16, 1.71)	1.42 (1.15, 1.84)	0.587
HDL-C (mmol/L)	0.68 (0.61, 0.78)	0.70 (0.63, 0.80)	0.63 (0.59, 0.74)	<0.001
LDL-C (mmol/L)	1.90 (1.81, 2.12)	1.86 (1.80, 2.12)	1.95 (1.82, 2.13)	0.145
UREA (mmol/L)	9 (6, 14)	7 (5, 12)	12 (8, 17)	<0.001
Cr (μmol/L)	71 (54, 125)	65 (52, 92)	105 (61, 169)	<0.001
UA (μmol/L)	237 (156, 323)	215 (150, 302)	279 (197, 388)	<0.001
GLU (mmol/L)	8.4 (6.1, 10.6)	7.7 (6.0, 9.8)	9.4 (6.9, 11.3)	<0.001
Ga (mmol/L)	1.89 (1.14, 2.12)	1.99 (1.20, 2.17)	1.71 (1.07, 1.96)	<0.001
Cl (mmol/L)	103 (100, 107)	103 (101, 106)	104 (100, 108)	0.405
K (mmol/L)	3.95 (3.57, 4.35)	3.99 (3.62, 4.32)	3.91 (3.49, 4.38)	0.161
Na (mmol/L)	138 (135, 142)	138 (136, 141)	139 (134, 144)	0.150
CTnI (ng/mL)	0.06 (0.02, 0.24)	0.03 (0.01, 0.13)	0.17 (0.05, 0.49)	<0.001
NT-proBNP (pg/mL)	2,310 (748, 6,180)	1,520 (532, 3,820)	4,495 (1,570, 11,384)	<0.001
TNF-α (pg/mL)	2.6 (2.0, 4.1)	2.4 (1.9, 3.5)	3.1 (2.1, 5.1)	<0.001
IL-6 (pg/mL)	73 (10, 557)	35 (6, 213)	358 (41, 1,958)	<0.001
LAC (mmol/L)	2.01 (1.66, 3.55)	1.83 (1.56, 2.35)	3.75 (2.20, 6.58)	<0.001
T (°C)	36.50 (36.50, 36.80)	36.50 (36.50, 36.80)	36.50 (36.50, 36.80)	0.746
R (breaths/min)	19.0 (17.0, 22.0)	18.6 (16.0, 20.0)	20.0 (18.0, 24.9)	<0.001
P (beats/min)	95 (81, 109)	93 (80, 107)	100 (85, 112)	0.015
DBP (mmHg)	121 (101, 134)	120 (100, 132)	123 (104, 135)	0.189
SBP (mmHg)	72 (60, 80)	72 (60, 80)	73 (61, 80)	0.440

RBC, red blood cell count; WBC, white blood cell count; LY, lymphocyte count; LY%, lymphocyte percentage; MO, monocyte count; MO%, monocyte percentage; NE, neutrophil count; NE%, neutrophil percentage; Hb, hemoglobin; HCT, hematocrit; MCV, mean corpuscular volume; RDW, red cell distribution width; PLT, platelet count; MPV, mean platelet volume; PDW, platelet distribution width; CRP, C-reactive protein; PCT, procalcitonin; PT, prothrombin time; TT, thrombin time; APTT, activated partial thromboplastin time; INR, international normalized ratio; FIB, fibrinogen; D-dimer, D-dimer; γ-GT, gamma-glutamyl transferase; ALT, alanine aminotransferase; AST, aspartate aminotransferase; ALP, alkaline phosphatase; LDH, lactate dehydrogenase; TBIL, total bilirubin; DBIL, direct bilirubin; UBIL, indirect bilirubin; TP, total protein; ALB, albumin; GLB, globulin; A/G, albumin-to-globulin ratio; TC, total cholesterol; TG, triglycerides; HDL-C, high-density lipoprotein cholesterol; LDL-C, low-density lipoprotein cholesterol; UREA, blood urea nitrogen; Cr, creatinine; UA, uric acid; GLU, glucose; Ga, calcium; Cl, chloride; K, potassium; Na, sodium; CTnI, cardiac troponin I; NT-proBNP, N-terminal pro-B-type natriuretic peptide; TNF-α, tumor necrosis factor-alpha; IL-6, interleukin-6; LAC, lactic acid; T, temperature; R, respiratory rate; P, pulse rate; DBP, diastolic blood pressure; SBP, systolic blood pressure.

### ML model and model interpretation

**Figure 2:**
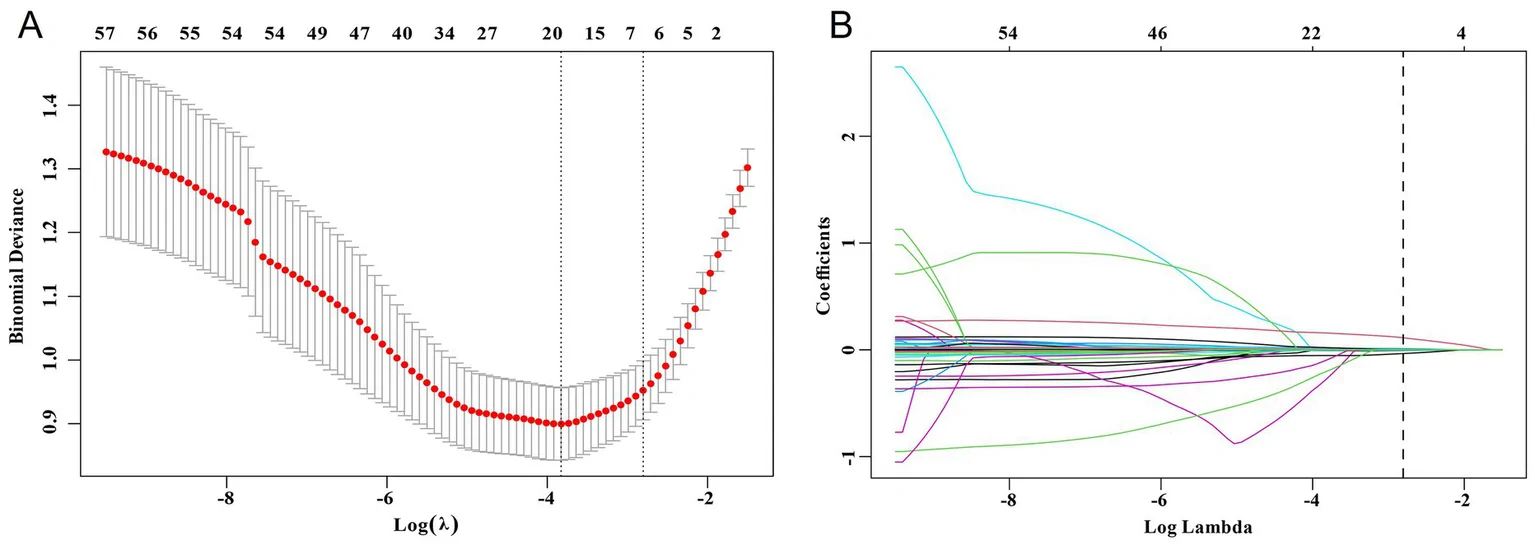
LASSO regression analysis. **(A)** Optimal *lambda* selection via 10-fold cross-validation. **(B)** Regularization paths showing coefficient shrinkage for all 35 candidate variables.

To reduce dimensionality and multicollinearity, LASSO regression was applied, resulting in the retention of six key variables: LAC, PLT, NE%, MO%, ALB and CRP (Figures 2A,B). Subsequent variance inflation factor (VIF) analysis confirmed the absence of multicollinearity among these variables [LAC (VIF 1.08), PLT (VIF 1.07), NE% (VIF 1.39), MO% (VIF 1.44), ALB (VIF 1.05) and CRP (VIF 1.11)].

**Figure 3:**
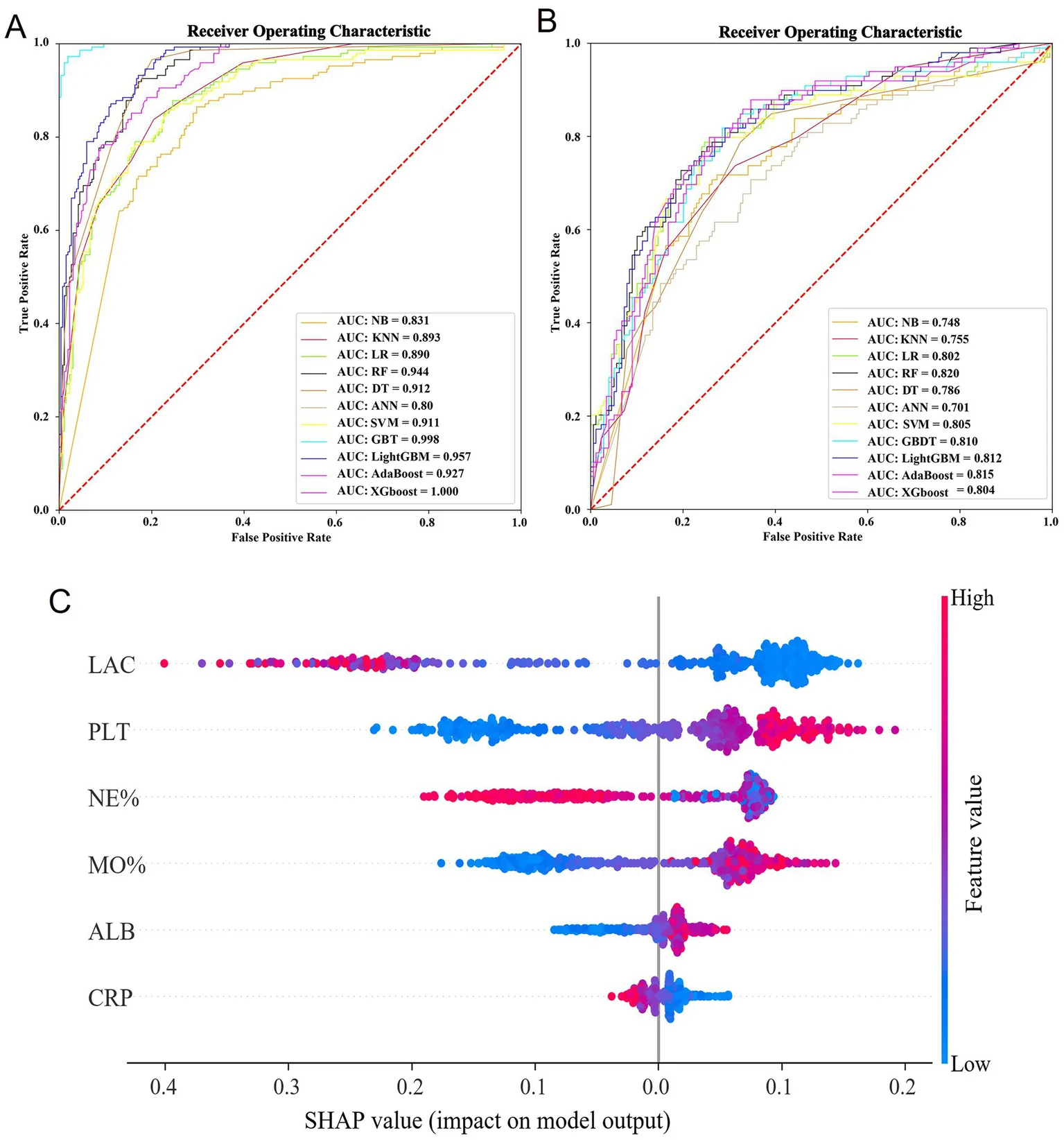
Construction and evaluation of the machine learning models. **(A)** ROC curves for 11 ML algorithms in the training cohort. **(B)** ROC curves for 11 ML algorithms in the validation cohort. **(C)** SHAP summary plot showing feature importance for the RF model.

**Figure 4:**
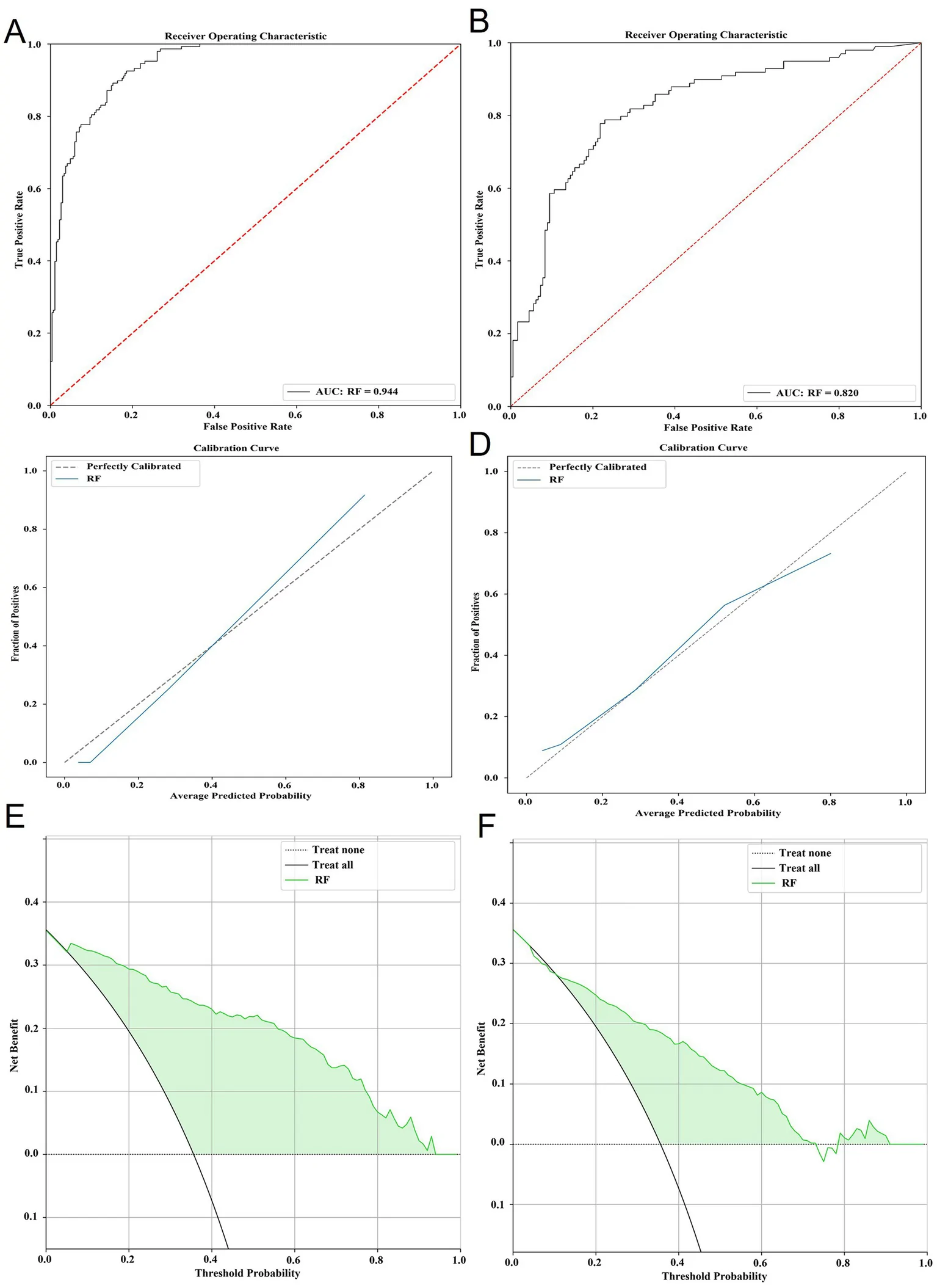
ROC curve, calibration curve, and DCA for the Random Forest (RF) algorithm utilizing the top six variables. **(A)** ROC curve in the training cohort. **(B)** ROC curve in the validation cohort. **(C)** Calibration curve in the training cohort. **(D)** Calibration curve in the validation cohort. **(E)** DCA in the training cohort. **(F)** DCA in the validation cohort.

In the training cohort, the four ML algorithms with the highest area under the curve (AUC) values were XGBoost (AUC = 1.000, 0.999–1.000), GBDT (AUC = 0.998, 0.993–1.000), LightGBM (AUC = 0.957, 0.934–1.000), RF (AUC = 0.944, 0.917–0.971). In the validation cohort, the top four algorithms ranked by AUC were RF (AUC = 0.820, 0.765–0.875), AdaBoost (AUC = 0.815, 0.759–0.871), LightGBM (AUC = 0.812, 0.756–0.868), GBDT (AUC = 0.810, 0.753–0.867) (Table 2) (Figures 3A,B). The 5-fold cross-validated AUC on the training cohort for XGBoost was 0.891 (0.858–0.923), compared with its training AUC of 1.000 (0.999–1.000) and validation AUC of 0.804 (0.747–0.861), confirming substantial optimism. For comparison, the final RF model showed stable performance (CV AUC: 0.898, 0.865–0.931; training AUC: 0.944, 0.917–0.971; validation AUC: 0.820, 0.765–0.875). The CV AUC for all 11 algorithms are provided in Supplementary Table 2. Given its consistently highest AUC performance across validation cohorts, RF was selected as the optimal model for this study. This model demonstrated excellent predictive performance, achieving an AUC of 0.944 in the training cohort and 0.820 in the validation cohort (Figures 4A,B). Calibration curves revealed strong agreement between the predicted probabilities generated by the Random Forest model and the actual observed outcomes (Figures 4C,D). Decision curve analysis (DCA) indicated that this Random Forest model yielded a higher net clinical benefit than either the “treat-all” or “treat-none” strategies (Figures 4E,F).

**Table 2:** Performance of the 11 machine learning algorithms in the training and validation cohorts for patients with septic shock.

Models	AUC	Accuracy	PPV	NPV	Sensitivity	Specificity	F1_score
Training cohort							
NB	0.831 (0.787–0.875)	0.755 (0.712–0.794)	0.612 (0.544–0.676)	0.896 (0.847–0.930)	0.851 (0.785–0.900)	0.703 (0.645–0.754)	0.761 (0.720–0.799)
KNN	0.893 (0.857–0.929)	0.806 (0.765–0.841)	0.719 (0.643–0.784)	0.856 (0.809–0.893)	0.743 (0.667–0.807)	0.840 (0.792–0.879)	0.806 (0.768–0.844)
LR	0.890 (0.853–0.927)	0.813 (0.773–0.847)	0.706 (0.633–0.769)	0.887 (0.841–0.920)	0.811 (0.740–0.866)	0.814 (0.763–0.856)	0.815 (0.778–0.851)
RF	0.944 (0.917–0.971)	0.859 (0.822–0.889)	0.751 (0.683–0.809)	0.938 (0.899–0.962)	0.899 (0.840–0.938)	0.836 (0.788–0.876)	0.861 (0.828–0.893)
DT	0.912 (0.879–0.945)	0.811 (0.770–0.845)	0.822 (0.739–0.883)	0.806 (0.759–0.847)	0.595 (0.514–0.670)	0.929 (0.892–0.954)	0.802 (0.760–0.841)
ANN	0.803 (0.756–0.850)	0.784 (0.742–0.821)	0.677 (0.602–0.744)	0.854 (0.805–0.892)	0.750 (0.675–0.813)	0.803 (0.751–0.846)	0.786 (0.747–0.825)
SVM	0.911 (0.878–0.944)	0.813 (0.773–0.847)	0.665 (0.599–0.725)	0.966 (0.931–0.983)	0.953 (0.906–0.977)	0.736 (0.680–0.785)	0.817 (0.781–0.853)
GBDT	0.998 (0.993–1.000)	0.976 (0.956–0.987)	0.960 (0.915–0.982)	0.985 (0.962–0.994)	0.973 (0.933–0.989)	0.978 (0.952–0.990)	0.976 (0.959–0.990)
LightGBM	0.957 (0.934–0.980)	0.882 (0.848–0.910)	0.780 (0.713–0.834)	0.958 (0.925–0.977)	0.932 (0.880–0.963)	0.855 (0.808–0.892)	0.884 (0.854–0.914)
XGBoost	1.000 (0.999–1.000)	0.998 (0.987–1.000)	1.000 (0.975–1.000)	0.996 (0.979–0.999)	0.993 (0.963–0.999)	1.000 (0.986–1.000)	0.998 (0.993–1.000)
AdaBoost	0.927 (0.897–0.957)	0.837 (0.798–0.869)	0.725 (0.655–0.785)	0.921 (0.879–0.949)	0.872 (0.808–0.916)	0.818 (0.767–0.859)	0.840 (0.805–0.874)
Validation cohort							
NB	0.748 (0.685–0.811)	0.727 (0.671–0.776)	0.600 (0.509–0.685)	0.816 (0.749–0.868)	0.697 (0.600–0.779)	0.743 (0.674–0.801)	0.730 (0.678–0.782)
KNN	0.755 (0.693–0.817)	0.712 (0.656–0.762)	0.587 (0.493–0.675)	0.793 (0.726–0.847)	0.646 (0.548–0.734)	0.749 (0.680–0.806)	0.715 (0.662–0.767)
LR	0.802 (0.744–0.860)	0.752 (0.698–0.799)	0.621 (0.533–0.702)	0.857 (0.793–0.904)	0.778 (0.686–0.848)	0.737 (0.668–0.796)	0.756 (0.706–0.805)
RF	0.820 (0.765–0.875)	0.745 (0.690–0.792)	0.604 (0.520–0.683)	0.875 (0.811–0.919)	0.818 (0.731–0.882)	0.704 (0.633–0.766)	0.750 (0.699–0.799)
DT	0.786 (0.727–0.845)	0.730 (0.675–0.779)	0.602 (0.512–0.685)	0.825 (0.759–0.876)	0.717 (0.622–0.796)	0.737 (0.668–0.796)	0.734 (0.683–0.784)
ANN	0.701 (0.635–0.767)	0.716 (0.660–0.766)	0.594 (0.499–0.683)	0.791 (0.724–0.845)	0.636 (0.538–0.724)	0.760 (0.692–0.816)	0.718 (0.665–0.769)
SVM	0.805 (0.748–0.862)	0.770 (0.717–0.815)	0.645 (0.556–0.724)	0.866 (0.804–0.911)	0.788 (0.697–0.857)	0.760 (0.692–0.816)	0.774 (0.725–0.820)
GBDT	0.810 (0.753–0.867)	0.745 (0.690–0.792)	0.600 (0.517–0.677)	0.891 (0.828–0.933)	0.848 (0.765–0.906)	0.687 (0.616–0.751)	0.750 (0.700–0.801)
LightGBM	0.812 (0.756–0.868)	0.759 (0.705–0.806)	0.654 (0.558–0.738)	0.822 (0.758–0.872)	0.687 (0.590–0.770)	0.799 (0.734–0.851)	0.760 (0.708–0.809)
XGBoost	0.804 (0.747–0.861)	0.752 (0.698–0.799)	0.606 (0.523–0.682)	0.904 (0.843–0.943)	0.869 (0.788–0.922)	0.687 (0.616–0.751)	0.757 (0.706–0.805)
AdaBoost	0.815 (0.759–0.871)	0.766 (0.713–0.812)	0.637 (0.550–0.716)	0.870 (0.808–0.914)	0.798 (0.708–0.865)	0.749 (0.680–0.806)	0.771 (0.722–0.817)

AUC, area under the curve; PPV, positive predictive value; NPV, negative predictive value; NB, Naive-Bayes; KNN, K-nearest Neighbor; LR, Logistic regression; RF, Random forest; DT, Decision tree; ANN, Artificial neural networks; SVM, Support vector machine; GBDT, Gradient boosting decision tree; LightGBM, Light gradient boosting machine; XGboost, Extreme gradient boosting; AdaBoost, Adaptive boosting.

To further clarify the contribution of each variable to the prediction model, SHAP method was applied to evaluate the RF model. The results indicated that LAC contributed the most to the model’s predictions, followed by PLT, NE%, MO%, ALB, CRP (Figure 3C).

Based on the above findings, we developed a web-based online calculator^1^ to enable other researchers to replicate our analysis and validate the model performance. This tool is intended for research purposes and has not yet undergone independent external testing.

Collectively, the results demonstrate that ML model based on the RF algorithm exhibited the best performance. We have established a simple, effective, and practical algorithm to assist clinicians in identifying high-risk patients.

## Discussion

Based on admission laboratory data, feature selection was performed using LASSO regression. Among 11 ML algorithms compared, RF model was selected as the best performer in the study, incorporating six variables: LAC, PLT, NE%, MO%, ALB and CRP. The RF algorithm demonstrated robustness against overfitting and an ability to capture nonlinear relationships and potential interactions among the six variables, which may offer advantages over traditional logistic regression in this context. This model may assist in predicting 30-day mortality in patients with septic shock, potentially supporting clinical decision-making. A web-based calculator was developed to facilitate its application and further validation.

When compared to recent ML-based prognostic models for septic shock, our RF model achieved a comparable or favorable performance (AUC 0.820), outperforming the TOPSIS-based Classification Fusion model’s internal validation AUC (0.733) (Wang et al., 2025) and comparable to Adaboost model’s internal validation AUC (0.822) (Wang et al., 2023). However, direct comparisons are challenging due to differences in study populations, predictor sets, and outcome definitions.

We observed that some complex ensemble algorithms, such as XGBoost and GBDT, exhibited a notable performance gap between the training and validation cohorts, a phenomenon indicative of overfitting. This is a recognized challenge when applying sophisticated ML algorithms to relatively small, high-dimensional clinical datasets. Notably, our final selected model, RF, demonstrated excellent resilience against overfitting, likely due to its bagging ensemble strategy and the effective dimensionality reduction achieved by LASSO regression. The stable and consistent performance of the RF model across both cohorts underscores the robustness of our selected predictor set and supports the clinical utility of our final model.

This study examined the relationship between LAC levels and patient prognosis. In the training cohort, admission LAC levels were significantly higher in non-survivors than in survivors (3.75 vs. 1.83 mmol/L, *p* < 0.001), consistent with previous evidence linking hyperlactatemia to increased mortality in septic shock (Zampieri et al., 2020; Chen et al., 2022). Mean lactate exceeded 5 mmol/L in non-survivors while remaining near 2.3 mmol/L in survivors across both cohorts, suggesting a lactate threshold of 2 mmol/L as a clinical warning indicator (Lee et al., 2021). Elevated LAC, reflecting tissue hypoxia and enhanced anaerobic glycolysis, arises directly from microcirculatory dysfunction and impaired mitochondrial function during septic shock (Wasyluk and Zwolak, 2021). Moreover, non-survivors showed not only elevated LAC but also more pronounced inflammatory responses, coagulation disorders, liver dysfunction, and renal deterioration. The correlations among these parameters indicate that elevated LAC is not an isolated event but rather a manifestation of the overall pathological state of multiple organs.

In addition to LAC, coagulation disorders also play a critical role in the pathophysiology of septic shock. In this study, the degree of thrombocytopenia was found to be significantly negatively correlated with 30-day mortality in patients with septic shock. This finding is consistent with previous studies, indicating that a decline in platelet count is an independent risk factor for poor prognosis in septic patients (Diao et al., 2023). The levels of PT, APTT, INR, and D-dimer in the non-survivor group were significantly prolonged or elevated (all *p* < 0.001), further confirming the presence of coagulation dysfunction in deceased patients. Mechanistically, during septic shock, pathogen-associated molecular patterns (PAMPs) and damage-associated molecular patterns (DAMPs) activate the coagulation cascade, leading to disseminated intravascular coagulation (DIC) and excessive platelet consumption (Toh et al., 2016). Concurrently, platelets promote microthrombus formation through activation mediated by surface Toll-like receptors (TLRs), thereby exacerbating organ ischemia (Zhang et al., 2019). Studies have demonstrated that septic plasma activates the NLRP6-TRIM21 complex within platelets via the TLR4/MyD88 signaling pathway, dynamically regulating TAB1 degradation and consequently limiting excessive NF-κB activation (Kim et al., 2019). Activated platelets further interact with neutrophils, promoting the formation of NETs (neutrophil ETs), which serve as a “scaffold” for thrombus formation, thereby establishing a self-amplifying thrombo-inflammatory network that ultimately exacerbates microcirculatory dysfunction and multi-organ ischemic injury (Zhang et al., 2023). Therefore, platelet count at admission not only reflects bone marrow reserve and consumption rate but also serves as a “microcosm” of microcirculatory failure, and should be considered a core indicator for early risk stratification assessment in septic shock.

This study found that the proportion of neutrophils was significantly increased in non-surviving patients, while the proportion of monocytes was significantly decreased. This pattern of elevated neutrophils and reduced monocytes essentially reflects an excessive early inflammatory response and subsequent immunosuppression in septic shock, which is consistent with the sepsis immunophenotypes reported in multiple previous studies (Lin et al., 2024; Rico-Feijoó et al., 2024). Activated neutrophils release NETs (neutrophil ETs), reactive oxygen species, and proteases, directly damaging the vascular endothelium and organ parenchyma (Xie et al., 2025). On the other hand, monocytes are significantly reduced due to increased apoptosis, leading to paralysis of the adaptive immune response, impaired pathogen clearance, and an increased risk of subsequent infection (Dong et al., 2023). In this study, the median monocyte percentage in non-surviving patients was 2.8%, primarily because during septic shock, the body is in a state of excessive inflammation, resulting in a massive release of neutrophils into the bloodstream. This causes neutrophils to dominate the differential white blood cell count, significantly “diluting” the monocyte percentage. As core cells involved in antigen presentation and immune regulation, the suppression of monocyte function plays a key role in sepsis-associated immunosuppression, and a sustained decrease in monocyte percentage is closely associated with poor prognosis.

This study found that the median albumin level in the non-survivor group was significantly lower than that in the survivor group (*p* < 0.001). This abnormal indicator reflects the combined effects of metabolic disorders and organ dysfunction in patients with septic shock. Regarding hypoalbuminemia, systemic inflammatory response leads to disruption of the capillary endothelial barrier, resulting in substantial leakage of albumin into the interstitial space. Concurrently, suppressed hepatic synthesis and a hypercatabolic state collectively contribute to the development of hypoalbuminemia (Piastra et al., 2026). Hypoalbuminemia not only affects colloid osmotic pressure and effective circulating volume but also alters the pharmacokinetics of antibiotics such as beta-lactams, increasing their volume of distribution. This may result in subtherapeutic concentrations, thereby elevating the risk of mortality (Idasiak-Piechocka et al., 2025).

In contrast, the CRP was significantly higher in the non-survivor group (*p* < 0.001), suggesting that elevated CRP is closely associated with a poor prognosis in patients with septic shock. CRP is an acute-phase protein synthesized by hepatocytes in response to stimulation by inflammatory cytokines such as IL-6, IL-1β, and TNF-α (Palalıoğlu et al., 2024). In septic shock, pathogens activate innate immunity and induce a robust inflammatory response, leading to a sharp increase in CRP levels (Long and Gottlieb, 2025). Furthermore, CRP directly participates in the host immune response by activating complement, promoting phagocytosis, and regulating cytokine release (Xu et al., 2025). Elevated CRP levels have been identified as a significant predictor of 28-day mortality in patients with sepsis and septic shock (Patnaik et al., 2024). Therefore, the combination of admission CRP levels with other inflammatory markers may facilitate early risk stratification in septic shock, thereby guiding anti-infective therapy and disease monitoring.

This study has several limitations. First, the data were derived from a single-center retrospective cohort, which may introduce selection bias and unmeasured confounding factors. Second, all laboratory indicators were collected only at the first time point upon admission, whereas these parameters dynamically change over time in septic shock patients. A single measurement may not fully capture the disease trajectory. Third, the exclusion of patients with ESRD on dialysis, Child-Pugh C cirrhosis, hematologic malignancy, and pregnancy/lactation may limit the generalizability of our findings to these specific subpopulations. Fourth, the lack of external validation limits the generalizability of the findings.

Our study presents a novel and interpretable machine learning framework for predicting 30-day mortality in septic shock, contributing to the growing body of literature that supports the utility of ML in critical care prognostication. While similar predictive models have been proposed, our work is distinguished by its focus on a highly accessible set of routine laboratory parameters and the development of a web-based tool for clinical translation.

In conclusion, by employing readily available laboratory indicators, we constructed a predictive model using machine learning algorithms, developed a web-based calculator, and provided a tool that may facilitate its translation into clinical practice. The model may be suitable for early risk stratification and the implementation of individualized treatment strategies, pending further validation. Future multicenter external validation is required to further confirm its generalizability.

## Data Availability

The raw data supporting the conclusions of this article will be made available by the authors, without undue reservation.

## Footnotes

1
https://jssrm-zj-6m6amcktxzcesfnabljknm.streamlit.app/
