# Supplementary material for: Development and validation of machine learning models for 30-day mortality prediction in septic shock
Source: Front Microbiol. 2026 Aug 28;17:1908032. doi: 10.3389/fmicb.2026.1908032 (PMC13561930; doi:10.3389/fmicb.2026.1908032)
Supplement: Supplementary file 1 [file Data_Sheet_1.PDF]

Supplementary Table 1. Comparison of baseline characteristics between the development cohort and validation cohort

Characteristic	Overall<br>N = 695	Development cohort<br>N = 417	Validation cohort<br>N = 278	p-value
**Sex (male)**	456 (65.6%)	263 (63.1%)	193 (69.4%)	0.084
**Age (yr)**	71 (59, 79)	70 (59, 79)	71 (61, 79)	0.656
**RBC (×1012/L)**	3.28 (2.83, 3.84)	3.31 (2.80, 3.84)	3.24 (2.85, 3.78)	0.517
**WBC (×10⁹/L)**	8 (5, 12)	8 (6, 13)	8 (5, 12)	0.835
**LY (×10⁹/L)**	0.94 (0.52, 1.38)	0.92 (0.53, 1.35)	0.94 (0.51, 1.39)	0.862
**LY%**	11 (6, 19)	11 (6, 19)	12 (5, 20)	0.843
**MO (×10⁹/L)**	0.44 (0.27, 0.69)	0.43 (0.26, 0.67)	0.45 (0.28, 0.69)	0.468
**MO%**	5.6 (3.0, 8.1)	5.5 (2.8, 8.0)	5.9 (3.2, 8.2)	0.350
**NE (×10⁹/L)**	6 (4, 11)	6 (4, 11)	7 (4, 10)	0.821
**NE%**	80 (69, 90)	80 (69, 90)	79 (69, 90)	0.571
**Hb(g/L)**	98 (85, 114)	98 (85, 115)	98 (85, 112)	0.947
**HCT (Ratio)**	30 (26, 35)	30 (26, 35)	30 (26, 35)	0.753
**MCV (fl)**	93 (90, 97)	93 (89, 96)	93 (90, 98)	0.219
**RDW (%)**	14.60 (13.40, 16.00)	14.60 (13.40, 16.10)	14.60 (13.40, 15.90)	0.669
**PLT (×10⁹/L)**	173 (93, 261)	172 (89, 255)	176 (103, 268)	0.506
**MPV**	10.82 ± 1.45	10.81 ± 1.40	10.84 ± 1.51	0.813
**PDW**	16.10 (13.20, 16.80)	16.10 (13.20, 16.80)	16.10 (13.20, 16.80)	0.666
**CRP (mg/L)**	77 (39, 132)	79 (40, 134)	77 (37, 131)	0.556
**PCT (ng/mL)**	3 (1, 14)	3 (1, 16)	3 (1, 12)	0.542
**PT(s)**	12.5 (11.5, 14.2)	12.5 (11.5, 14.2)	12.5 (11.5, 14.4)	0.765
**TT(s)**	17 (16, 19)	17 (16, 18)	17 (16, 19)	0.975
**APTT(s)**	29 (26, 35)	30 (27, 35)	29 (26, 36)	0.907
**INR (Ratio)**	1.09 (1.00, 1.25)	1.09 (1.00, 1.25)	1.09 (1.00, 1.27)	0.811
**FIB(g/L)**	4.70 (3.36, 6.18)	4.67 (3.40, 6.09)	4.79 (3.35, 6.22)	0.801
**D-D (mg/L)**	3.9 (2.0, 7.7)	4.0 (2.0, 7.8)	3.8 (2.0, 7.6)	0.496
**γ-GT(U/L)**	52 (28, 103)	51 (27, 102)	55 (31, 104)	0.141
**ALT(U/L)**	25 (15, 48)	25 (14, 44)	26 (17, 54)	0.040
**AST(U/L)**	32 (20, 58)	30 (20, 57)	34 (21, 60)	0.063
**ALP(U/L)**	97 (72, 137)	97 (73, 141)	98 (71, 130)	0.464
**LDH(U/L)**	240 (186, 360)	240 (185, 358)	241 (188, 363)	0.813
**TBIL (μmol/L)**	15 (10, 25)	14 (10, 22)	15 (10, 29)	0.143
**DBIL (μmol/L)**	5 (3, 12)	5 (3, 10)	5 (3, 14)	0.463
**UBIL (μmol/L)**	7 (4, 12)	7 (4, 11)	7 (4, 13)	0.321
**TP (g/L)**	57 (52, 63)	58 (52, 64)	56 (52, 62)	0.113
**ALB (g/L)**	30.1 ± 4.9	30.4 ± 5.0	29.7 ± 4.8	0.088
**GLB (g/L)**	26.9 (23.4, 31.4)	27.1 (23.5, 31.3)	26.4 (23.3, 31.4)	0.465

Characteristic	Overall<br>N = 695	Development cohort<br>N = 417	Validation cohort<br>N = 278	p-value
**A/G**	1.12 (0.95, 1.29)	1.13 (0.95, 1.29)	1.10 (0.96, 1.29)	0.605
**TC (mmol/L)**	2.90 (2.84, 3.24)	2.90 (2.83, 3.14)	2.92 (2.84, 3.40)	0.107
**TG (mmol/L)**	1.43 (1.15, 1.79)	1.41 (1.16, 1.74)	1.46 (1.15, 1.83)	0.397
**HDL-C (mmol/L)**	0.68 (0.61, 0.78)	0.68 (0.61, 0.78)	0.68 (0.61, 0.81)	0.985
**LDL-C (mmol/L)**	1.92 (1.81, 2.17)	1.90 (1.81, 2.12)	1.94 (1.81, 2.24)	0.089
**UREA (mmol/L)**	9 (6, 14)	9 (6, 14)	9 (6, 15)	0.659
**Cr (µmol/L)**	72 (53, 130)	71 (54, 125)	73 (53, 142)	0.741
**UA (µmol/L)**	236 (156, 328)	237 (156, 323)	234 (154, 339)	0.827
**GLU (mmol/L)**	8.4 (6.0, 10.6)	8.4 (6.1, 10.6)	8.3 (5.8, 10.5)	0.363
**Ga(mmol/L)**	1.90 (1.13, 2.11)	1.89 (1.14, 2.12)	1.92 (1.13, 2.11)	0.835
**Cl (mmol/L)**	103 (100, 107)	103 (100, 107)	103 (99, 107)	0.198
**K (mmol/L)**	3.96 (3.57, 4.35)	3.95 (3.57, 4.35)	3.97 (3.56, 4.36)	0.701
**Na (mmol/L)**	138 (135, 142)	138 (135, 142)	138 (134, 141)	0.046
**CTnI (ng/mL)**	0.06 (0.02, 0.24)	0.06 (0.02, 0.24)	0.06 (0.02, 0.24)	0.621
**NT-proBNP (pg/mL)**	2,310 (694, 6,300)	2,310 (748, 6,180)	2,308 (663, 6,870)	0.852
**TNF-α(pg/mL)**	2.7 (2.0, 4.1)	2.6 (2.0, 4.1)	2.7 (2.0, 4.1)	0.390
**IL-6(pg/mL)**	85 (9, 552)	73 (10, 557)	103 (8, 514)	0.843
**LAC (mmol/L)**	2.10 (1.67, 3.54)	2.01 (1.66, 3.55)	2.22 (1.69, 3.44)	0.410
**T (°C)**	36.50 (36.50, 36.80)	36.50 (36.50, 36.80)	36.60 (36.50, 36.90)	0.152
**R (breaths/min)**	19.0 (17.0, 22.0)	19.0 (17.0, 22.0)	19.0 (17.0, 22.0)	0.719
**P(beats/min)**	95 (82, 110)	95 (81, 109)	96 (86, 112)	0.109
**DBP (mmHg)**	122 (103, 133)	121 (101, 134)	122 (104, 133)	0.597
**SBP (mmHg)**	72 (61, 80)	72 (60, 80)	73 (61, 80)	0.994

RBC: Red Blood Cell Count; WBC: White Blood Cell Count; LY: Lymphocyte Count; LY%: Lymphocyte Percentage; MO: Monocyte Count; MO%: Monocyte Percentage; NE: Neutrophil Count; NE%: Neutrophil Percentage; Hb: Hemoglobin; HCT: Hematocrit; MCV: Mean Corpuscular Volume; RDW: Red Cell Distribution Width; PLT: Platelet Count; MPV: Mean Platelet Volume; PDW: Platelet Distribution Width; CRP: C-reactive Protein; PCT: Procalcitonin; PT: Prothrombin Time; TT: Thrombin Time; APTT: Activated Partial Thromboplastin Time; INR: International Normalized Ratio; FIB: Fibrinogen; D-Dimer: D-Dimer; γ-GT: Gamma-Glutamyl Transferase; ALT: Alanine Aminotransferase; AST: Aspartate Aminotransferase; ALP: Alkaline Phosphatase; LDH: Lactate Dehydrogenase; TBIL: Total Bilirubin; DBIL: Direct Bilirubin; UBIL: Indirect Bilirubin; TP: Total Protein; ALB: Albumin; GLB: Globulin; A/G: Albumin-to-Globulin Ratio; TC: Total Cholesterol; TG: Triglycerides; HDL-C: High-Density Lipoprotein Cholesterol; LDL-C: Low-Density Lipoprotein Cholesterol; UREA: Blood Urea Nitrogen; Cr: Creatinine; UA: Uric Acid; GLU: Glucose; Ga: Calcium; Cl: Chloride; K: Potassium; Na: Sodium; CTnI: Cardiac Troponin I; NT-proBNP: N-terminal pro-B-type Natriuretic Peptide; TNF-α: Tumor Necrosis Factor-alpha; IL-6: Interleukin-6; LAC: Lactic Acid; T: Temperature; R: Respiratory Rate; P: Pulse Rate; DBP: Diastolic Blood Pressure; SBP: Systolic Blood Pressure.

Normally distributed continuous variables are presented as mean ± standard deviation; non-normally distributed continuous variables are presented as median (interquartile range).
