# Supplementary material for: Development and validation of machine learning models for 30-day mortality prediction in septic shock
Source: Front Microbiol. 2026 Aug 28;17:1908032. doi: 10.3389/fmicb.2026.1908032 (PMC13561930; doi:10.3389/fmicb.2026.1908032)
Supplement: Supplementary file 2 [file Data_Sheet_2.DOCX]

**Supplementary Table 2** Performance of the eleven machine learning algorithms in the training cohort, training (5-fold cross-validation) and the validation cohort.

| **models** | **Training AUC** | **Training (5‑fold CV) AUC** | **Validation AUC** |
| --- | --- | --- | --- |
| **NB** | 0.831 (0.787-0.875) | 0.879 (0.860–0.898) | 0.748 (0.685-0.811) |
| **KNN** | 0.893 (0.857-0.929) | 0.871 (0.826–0.916) | 0.755 (0.693-0.817) |
| **LR** | 0.890 (0.853-0.927) | 0.877 (0.838–0.916) | 0.802 (0.744-0.860) |
| **RF** | 0.944 (0.917-0.971) | 0.898 (0.865–0.931) | 0.820 (0.765-0.875) |
| **DT** | 0.912 (0.879-0.945) | 0.870 (0.835–0.905) | 0.786 (0.727-0.845) |
| **ANN** | 0.803 (0.756-0.850) | 0.892 (0.858–0.926) | 0.701 (0.635-0.767) |
| **SVM** | 0.911 (0.878-0.944) | 0.891 (0.850–0.932) | 0.805 (0.748-0.862) |
| **GBDT** | 0.998 (0.993-1.000) | 0.889 (0.856–0.923) | 0.810 (0.753-0.867) |
| **LightGBM** | 0.957 (0.934-0.980) | 0.889 (0.862–0.917) | 0.812 (0.756-0.868) |
| **XGBoost** | 1.000 (0.999-1.000) | 0.950 (0.858–0.923) | 0.804 (0.747-0.861) |
| **AdaBoost** | 0.927 (0.897-0.957) | 0.884 (0.852–0.917) | 0.815 (0.759-0.871) |

AUC: area under the curve, PPV: positive predictive value, NPV: negative predictive value, NB: Naive-Bayes, KNN: K-nearest Neighbor, LR: Logistic regression, RF: Random forest, DT: Decision tree, ANN: Artificial neural networks, SVM: Support vector machine,GBDT: Gradient boosting decision tree, LightGBM: Light gradient boosting machine, XGboost: Extreme gradient boosting, AdaBoost: Adaptive boosting
